# If we are all cultural Darwinians what’s the fuss about? Clarifying recent disagreements in the field of cultural evolution

**DOI:** 10.1007/s10539-015-9490-2

**Published:** 2015-06-03

**Authors:** Alberto Acerbi, Alex Mesoudi

**Affiliations:** Department of Archaeology and Anthropology, University of Bristol, 43 Woodland Road, Bristol, BS8 1UU UK; Department of Anthropology, Durham University, Dawson Building, South Road, Durham, DH1 3LE UK; Philosophy & Ethics, School of Innovation Sciences, Eindhoven University of Technology, Den Dolech 2, Eindhoven, The Netherlands; Human Biological and Cultural Evolution Group, Department of Biosciences, College of Life and Environmental Sciences, University of Exeter, Penryn Campus, Penryn, Cornwall, TR10 9FE UK

**Keywords:** Cultural attraction, Cultural attractors, Cultural evolution, Cultural transmission

## Abstract

Cultural evolution studies are characterized by the notion that culture evolves accordingly to broadly Darwinian principles. Yet how far the analogy between cultural and genetic evolution should be pushed is open to debate. Here, we examine a recent disagreement that concerns the extent to which cultural transmission should be considered a preservative mechanism allowing selection among different variants, or a transformative process in which individuals recreate variants each time they are transmitted. The latter is associated with the notion of “cultural attraction”. This issue has generated much misunderstanding and confusion. We first clarify the respective positions, noting that there is in fact no substantive incompatibility between cultural attraction and standard cultural evolution approaches, beyond a difference in focus. Whether cultural transmission should be considered a preservative or reconstructive process is ultimately an empirical question, and we examine how both preservative and reconstructive cultural transmission has been studied in recent experimental research in cultural evolution. Finally, we discuss how the relative importance of preservative and reconstructive processes may depend on the granularity of analysis and the domain being studied.

## Introduction

Cultural evolution is a vibrant, interdisciplinary, and increasingly productive scientific framework that aims to provide a naturalistic and quantitative explanation of culture, in both human and non-human species (Mesoudi 2011; Richerson and Christiansen 2013). ‘Culture’ is commonly defined as the body of information that is transmitted from individual to individual via social learning (rather than genetically), and colloquially includes such phenomena as attitudes, beliefs, knowledge, skills, customs and institutions. Inspired by pre-existing population genetics tools, the mathematical models of cultural dynamics developed by Cavalli-Sforza and Feldman (1981) and Boyd and Richerson (1985) first established that cultural change can be modelled as an evolutionary process yet one that is not slavishly identical in its details to genetic evolution. Today, while maintaining a solid modelling core (e.g. Kendal et al. 2009; Rendell et al. 2010; Aoki et al. 2011; Lewis and Laland 2012; Aoki et al. 2012; Kempe et al. 2014), a wide range of methodologies are used in the field of cultural evolution, including phylogenetic analysis (e.g. Gray and Jordan 2000; Tehrani and Collard 2002; Lycett 2009; Currie et al. 2010; Tehrani 2013; O’Brien et al. 2014), laboratory experiments (e.g. Mesoudi et al. 2006; Caldwell and Millen 2008; Mesoudi and O’Brien 2008; Kirby et al. 2008; Morgan et al. 2012; Derex et al. 2013; Muthukrishna et al. 2014; Tamariz et al. 2014), ethnographic field studies (e.g. Guglielmino et al. 1995; Henrich and Henrich 2010; Mathew and Boyd 2011; Hewlett et al. 2011; Demps et al. 2012; Kline et al. 2013), quantitative analysis of pre-historical, historical, and contemporary datasets (e.g. Shennan and Wilkinson 2001; Henrich 2001; Kline and Boyd 2010; Collard et al. 2011; Turchin et al. 2013; Acerbi and Bentley 2014; Beheim et al. 2014), and comparative studies of culture across species (Whiten et al. 1999; Laland et al. 2011; Dean et al. 2012). Although varied in methodology and topic, these studies are united by the notion that culture evolves according to broadly Darwinian principles.

In parallel with this approach, a group of cognitive anthropologists have advanced a similar project aiming towards naturalistic explanations of culture, mainly focusing on the role that cognitive factors play in the transmission and transformation of cultural representations (Sperber 1996; Atran 1998; Boyer 2001; Sperber and Hirschfeld 2004). This approach has generated findings using laboratory experiments (e.g. Boyer and Ramble 2001; Barrett and Nyhof 2001; Norenzayan et al. 2006; Fessler et al. 2014) and analyses of historical (e.g. Nichols 2002; Norenzayan et al. 2006; Morin 2013) and cross-cultural (e.g. Atran 1998) datasets. The two approaches initially developed separately and, despite a series of attempts at seeking common ground (Henrich and Boyd 2002; Claidière and Sperber 2007; Sperber and Claidière 2008; Henrich et al. 2008), there is remaining disagreement (see e.g. Claidière et al. 2014).

This disagreement rests, at a general level, in a different view of cultural transmission. For the standard cultural evolution approach, typified by Boyd, Richerson, Henrich and others, it is common to think of cultural evolution as a process of selection between different variants (e.g. beliefs, ideas or artefacts) or models (referring to people from whom one can copy). When deciding a name for a newborn, for example, one chooses from a pool of variants—the existing names in the population—and the individual-level processes of selection determine the success, at the population-level, of the variants. Cultural transmission has relatively high fidelity, and selection between faithfully transmitted variants plays an important role in determining cultural trajectories.

Sperber, Claidière, Atran, Boyer and colleagues, instead, argue that in the vast majority of cases cultural traits are neither properly copied or selected, but reconstructed each time an instance of transmission happens. The permanence of some cultural traits occurs not due to high fidelity cultural transmission but instead due to the existence of stable “cultural attractors” (Sperber 1996). For example, in an oral transmission of a story, say Cinderella, it is highly unlikely the story will be repeated verbatim at each passage. Still, some defining features, say the pumpkin coach or the wicked stepmother, perhaps because they are particularly memorable, will act as attractors, and will be repeated (‘reconstructed’) each time by different narrators. Cultural transmission here has relatively low fidelity, and non-random distortions and reconstructions play an important role in maintaining cultural diversity and stability.

This general divergence has a series of consequences, ranging from what are considered the most important or interesting factors to take into account when explaining the permanence and diffusion of cultural traits (cognitive transformation of representations for Sperber and colleagues, interaction of simple decision-making biases with populational dynamics for the standard cultural evolution approach) to how far the analogy between cultural and biological evolution should be pushed (less for the former than for the latter approach).

In our view, there is no real conflict between the two approaches, besides a focus on different aspects of cultural evolution. Yet much confusion and disagreement seems to surround these issues, despite occasional claims of reconciliation and compatibility. The aim of this paper is to clarify the two positions, identifying areas of common ground and genuine disagreement, and suggest how cultural evolution research should best proceed.

In the following sections, we will first outline the basic tenets of the two approaches, and then move to defend the usefulness of a *narrow* notion of cultural attraction, as opposed to the broader notion proposed by Sperber and colleagues (e.g. Sperber 1996; Claidière et al. 2014), that we define as *extended*. The narrow notion is applied when cultural transmission is a mainly reconstructive process (as in the Cinderella example), and is contrasted with cases in which cultural transmission is a mainly preservative process and preferential selection is the most important driver of cultural dynamics (as in the baby names example). We will discuss how narrow attraction and preferential selection are better viewed as two extremes of a continuum (see also El Mouden et al. 2014) in which the relative importance of preservative and reconstructive processes is ultimately an empirical question, varying in different cultural domains, and depending on the granularity of the analysis. Finally, one weakness of current debates is the reliance on fictional thought experiments of unclear real-world relevance, or formal models with assumptions that can be manipulated to support either position. We therefore discuss actual empirical examples in which attraction has been considered, drawing on the now-vast cultural evolution literature.

## Two approaches for explaining culture

### The standard cultural evolution approach

The notion that human culture ‘evolves’ in a manner similar to the way in which species evolve has a long history. Darwin (1871) himself, in the Descent of Man, drew on the work of historical linguists who were already constructing informal evolutionary trees of language families (see van Whine 2005). Although the idea of cultural evolution sporadically emerged during the early to mid 20th century, such as in the work of psychologist Donald Campbell (e.g. Campbell 1965), it was not until the 1970s and 1980s that a quantitative science of cultural evolution was established, primarily by Cavalli-Sforza and Feldman (1981) and Boyd and Richerson (1985). These researchers began with classic models of population genetics developed within evolutionary biology in the early 20th century by Fisher, Haldane, Wright and others. Population genetic models are essentially mathematical formalisations of the evolutionary process outlined by Darwin, with added assumptions about genetic inheritance, genetic mutation and the like that subsequent experimentalists added to Darwin’s basic theory. They provide a way of exploring how the events in the lives of individuals—survival, reproduction, the rules of inheritance, etc.—scale up over successive generations and in large populations. Mathematical formalism proved far superior to verbal descriptions, and population genetic models resolved all manner of confusion over issues such as how particulate inheritance of discrete units (genes) could be reconciled with the apparent blending of continuous traits, and how natural selection can yield significant evolutionary change without any kind of Lamarckian transformation (see Mesoudi 2011, pp. 47–51).

Cavalli-Sforza and Feldman (1981) and Boyd and Richerson (1985) aimed to do the same for culture: to adapt population genetic models to be suitable for cultural change (see Mesoudi 2011, Ch 3 for an accessible overview of this work). These models made assumptions about the lives of individuals—who they learn from, who they pass cultural traits onto, the rules of cultural inheritance etc.—and then explore the long-term, population-level consequences of these events over many generations and in large, often structured populations. While they adopted the same mathematical tools as used in biology, they were careful not to import assumptions regarding genetic evolution that are unlikely to apply to cultural evolution. For example, Cavalli-Sforza and Feldman (1981) modelled the consequences of not just vertical cultural transmission (learning from biological parents) but also oblique cultural transmission (learning from unrelated members of the parental generation) and horizontal cultural transmission (learning from peers), as well as specific forms of the latter such as one-to-many transmission (typical of mass media). Boyd and Richerson (1985) modelled conformist cultural transmission (preferentially adopting the majority behaviour in the population) and model-based cultural transmission (preferentially learning from particularly high status or prestigious individuals), which again have no clear parallel in biological evolution. Boyd and Richerson (1985) also explored the conditions under which cultural transmission biases such as conformity might be favoured by genetic evolution and the two-way interaction between genetic and cultural evolution, or what is known as gene-culture coevolution (or dual-inheritance).

Of most relevance to the present paper are Boyd and Richerson’s (1985) models of ‘guided variation’ and ‘direct’ (or ‘content’) bias. Guided variation occurs when individuals transform cultural variants in a non-random (perhaps genetically adaptive) direction due to trial and error learning or some higher-level cognitive inductive process, and then pass on this modified trait to others in an unbiased (random) manner. The cause of change here lies within the individual, rather than with any populational process of selection between different variants or different people (because transmission is unbiased). Boyd and Richerson (1985) show, as one would intuitively expect, that if everyone transforms traits in the same direction, then the population quickly converges on this individually-favoured trait. Note that there is nothing like selection going on in this model. Indeed, it could instead be described as a form of Lamarckism, with evolutionary change driven by non-random modifications made by individuals.

Guided variation can be contrasted with direct bias (Boyd and Richerson 1985; later renamed ‘content bias’ in Richerson and Boyd 2005), where individuals survey all traits in the population, individually evaluate them (based, for example, on whether they are more effective or efficient than existing traits, or whether they fit with pre-existing cognitive biases), and preferentially adopt certain traits over other traits. Unlike guided variation, content bias *is* selection-like, because it does not itself change or transform the trait in any way, it just changes the trait’s frequency in the population. Hence the strength of content bias (like the strength of natural selection) depends on the amount of variation that is in the population. Guided variation, in contrast, works irrespective of variation in the population. The aforementioned model-based and conformist biases are similar to content bias in being selection-like, i.e. particular traits (e.g. those held by successful or prestigious individuals, or those exhibited by the majority) are preferentially adopted, with no change to those traits themselves other than their frequency. Note that Boyd and Richerson (1985) themselves, in that and subsequent work, have been reluctant to label the latter processes as ‘cultural selection’ preferring the term ‘biased transmission’ (e.g. Richerson and Boyd 2005, p. 69). Following Cavalli-Sforza and Feldman (1981) and Mesoudi (2011) we nevertheless treat these as equivalent to cultural selection, given that they involve the selection of particular traits over others with no modification of those traits. Guided variation, however, is unambiguously non-selection-like, a point we return to later.

### Sperberian cultural attraction

In parallel with the development of the standard cultural evolutionary approach, Dan Sperber and a group of cognitive anthropologists and psychologists developed a program of naturalistic explanation of cultural phenomena mainly aimed, at least initially, at recognizing the importance of universal cognitive factors in shaping cultural differences and regularities (Sperber 1996; Sperber and Hirschfeld 2004). In contrast to mainstream socio-cultural anthropology, which in recent decades has largely shied away from cross-cultural comparisons, Sperber and collaborators emphasised the existence of trans-cultural regularities in domains such as religion (Boyer 2001), social classification (Hirschfeld 1998), and folk-biology (the way in which people reason about plants and animal: Atran 1998).

The explanation for these regularities can be found in how representations interact with universal cognitive constraints. Again in contrast to the majority of socio-cultural anthropology, this approach endorsed a vision of cognitive architecture in which cognition is endowed with a rich, genetically-determined structure, that influences cultural processes in a meaningful way. In particular, Sperber defended what is known as the massive modularity hypothesis (Sperber 1996), that is, the idea that the mind is composed of a multitude of information-processing mechanisms that operate autonomously or quasi-autonomously on specific domains (Carruthers 2006). In this way, Sperber and colleagues’ approach can be aligned to Tooby and Cosmides’ brand of evolutionary psychology (Tooby and Cosmides 1992; Pinker 2010), which similarly advocates the existence of domain-specific, pan-human, cognitive modules that evoke similar behaviours in response to similar environmental cues.

In the case of religion (Boyer 2001), for example, successful supernatural concepts such as gods or angels are characterised by a combination of (1) consistency to intuitive, universal expectations—produced by our domain-specific cognitive architecture—about intentional agents (gods are jealous, they see our actions, they punish and reward, etc.) and (2) a few relevant violations of those expectations (gods are immortal and omnipresent, they can read our thoughts, etc.). This combination makes these minimally counter-intuitive entities easier to recall, as well as more likely to be spontaneously recreated. This in turn favours the success and the stability of those cultural systems, like religions—but also folk tales—in which they are present. Minimally counter-intuitive entities function as “cultural attractors”. These predictions have been supported by laboratory experiments showing that minimally counterintuitive representations are better remembered and passed on (Barrett and Nyhof 2001; Boyer and Ramble 2001), and analyses of actual folk tales showing that successful tales contain an optimal number of counterintuitive elements (Norenzayan et al. 2006).

As part of this theoretical framework, Sperber (1996) challenged the standard cultural evolution approach, in particular the adequacy of the “selectionist” character of its decision-making biases, and it is this challenge that we focus on in the present paper. According to Sperber, describing cultural evolution as a process of selection between different alternatives is quite misleading: cultural traits do not replicate in the process of transmission, instead they are transformed and reconstructed each time. A proper process of selection, such as natural selection as it operates on genetic replicators, needs to be sustained by low rates of mutation that are simply impossible to achieve in the case of human cultural transmission.

How is it, then, that Cinderella is still here with us? Sperber and colleagues do not deny the macrostability of culture, that is, the fact that some traditions are successfully preserved in time and space, often over many generations. However, they reject the assumption that this happens because the transmission of traits at the individual level is highly faithful. Instead, the transformations that occur at each transmission event are, in the majority of cases, non-random. Amongst all possible reconstructions of Cinderella, some of them, perhaps the ones featuring a pumpkin coach or ugly stepsisters, are more likely than others to happen. The idea of a “cultural attractor” rests on the assumption that transformations are not equally probable, and instead are biased in some direction. If, as Sperber suggests, one thinks of all possible transformations in a space of possibilities, probabilities will tend to cluster in some points of this space.

To say that an attractor exists, Sperber adds, is not an explanation for a given cultural phenomenon, but it is a way “to suggest the kind of causal explanations to be sought: namely, the identification of genuine causal factors that bias micro-transformations” (Sperber 1996, p. 112), or “factors of attraction”. As noted above, the factors of attraction identified typically reflect g domain-specific features of cognition. Consider again Cinderella. The pumpkin coach might be a good example of a minimally counter-intuitive element, particularly likely to be retained (see Norenzayan et al. 2006 for an analysis of folk tales, including Cinderella). The presence of the wicked stepmother, meanwhile, has been argued to have its origins in kin selection (Daly and Wilson 1999), where child abuse and, in extreme cases, infanticide are more likely to occur between genetically unrelated people, such as stepparents and stepchildren. In both of these cases, then, cultural stability arises not through high-fidelity transmission but because pumpkin coaches and non-kin-directed child abuse are salient cultural attractors that are particularly likely to be remembered and reconstructed in successive retellings.

### Clarifying ‘cultural attraction’

More recently, the idea of cultural attraction has been reassessed by Claidière et al. (2014). First, they emphasise that *factors of attraction are not exclusively cognitive*. While this has been clearly specified from Sperber’s initial presentation (factors of attraction were classified as ‘psychological’ and ‘ecological’ in Sperber 1996), the existence of non-cognitive factors of attraction is often overlooked, and attractors are usually labelled “cognitive attractors” (see e.g. Henrich et al. 2008). This confusion may have arisen because cultural attraction advocates themselves sometimes suggest this interpretation. For example, Claidière and Sperber (2007) write, “the idea of attraction […] aims at explaining the relative prevalence and stability of cultural content as a function of *properties of the content themselves*” (p. 91, our italics). However, Claidière et al. (2014) make it clear that factors of attraction encompass everything that creates attractors in the space of possibilities of transformations. Given that this has generated confusion in the literature, we think it is worth clearly pointing it out here.

Second, and potentially more significantly, Claidière et al. (2014) clarify how attraction encompasses all instances of cultural change (see also Sperber 1996). Because factors of attraction encompass everything that creates attractors in the space of possibilities of transformations, Claidière et al. (2014) therefore argue that this also includes perfect replication, which supports selection processes. Selection is therefore considered a special case of attraction. The consequence of this interpretation is that “attraction” is synonymous with *any* directional change in cultural evolution, whether due to transformations of cultural traits during transmission or not. Processes like content bias, conformity, or prestige-based bias, which are usually seen as distinct from attraction (Henrich et al. 2008), are considered by Claidière et al. (2014) to be examples of attraction. Again, much confusion surrounds this issue. In the same paper it is written, for example, that “the constructive processes we discussed above may tend to *transform* different inputs in similar ways (rather than randomly), and in doing so cause the outputs to tendentially converge upon particular types, called attractors. *This tendency is called cultural attraction*” (Claidière et al. 2014, our italics). Elsewhere, Claidière and Sperber (2007) explicitly talk of two “kinds of phenomena—distribution-based transmission biases and content-based attraction” (p. 91). The example used by Claidière and Sperber (2007) is smoking: initially naïve individuals copy the smoking rate of a light smoker (selection), and then independently transform this number towards one of two attractors, either zero cigarettes or 25 cigarettes (attraction). Selection and attraction are clearly separate and different processes, as we have presented them in the previous section.

However, from the examples included in Claidière et al. (2014), it appears that all directional processes of cultural evolution would fall under the general umbrella of ‘attraction’ (possibly excluding cultural drift, a process analogous to genetic drift in which the success of cultural traits is simply due to chance, as some traits are observed, or transmitted, more than others for random reasons - see Bentley et al. 2004). We therefore call this the *extended* concept of attraction, which encompasses any directional (non-random) cultural change. We contrast this with a *narrow* concept of attraction, which refers only to the transformative, non-selective processes in cultural transmission, and which seems closer to Claidière and Sperber’s (2007) definition, when they explicitly model the relative strength of selection and attraction in cultural transmission. In the rest of our paper we restrict cultural attraction to the narrow sense, because this seems to capture the genuine theoretical disagreement: the extent to which cultural evolution is influenced by individual transformation or by selection-like processes.

We note first, however, that the extended concept of cultural attraction seems little different to the notion of cultural evolution presented by Cavalli-Sforza and Feldman (1981); Boyd and Richerson (1985), and others, as discussed above. Claidière et al. (2014) extended notion of attraction incorporates reconstructive, transformative processes (which we call narrow attraction), as well as selection-like model-based, frequency-dependent and content-based biases that rest on higher fidelity cultural transmission. Cultural evolution as presented by Boyd and Richerson (1985), too, contains the transformative process of guided variation, which in our view seems identical to narrow attraction, as well as selection-like model-based and content-based biases, as described in Sect. 2.1 above. If extended cultural attraction is nothing more than cultural evolution, then we see little need to adopt extended cultural attraction as a concept, because these processes have already been modelled and extensively studied under the name ‘cultural evolution’.

### Modelling (narrow) attraction and selection

Henrich and Boyd (2002) used a mathematical model to show that, when both selection and attraction are present, the outcomes of the process are determined by selection alone. A verbal description of the model is as follows (see also Henrich et al. 2008): consider a continuous mental representation, for example, concerning what governs the moon’s behaviour. At one extreme (attractor-0) the moon is considered an intentional agent, and its behaviour can be explained using folk psychology (the moon wants to hide under the horizon, it changes shape to communicate something to us, etc.). At the other extreme (attractor-1) the moon is simply considered to be a “big rock”, lacking motivations and goals, and physical laws such as gravitation determine its behaviour. Ideas between the two attractors are possible (one can think that the movement of the moon is determined by gravitation, but the shape is related to the moon’s emotions), but, as the two extremes are more internally coherent, transformation of the representations will not be equiprobable, and will tend towards one or the other attractor. This is (narrow) cultural attraction.

Now for selection: imagine that individuals with a mental representation close to attractor-1 are more successful, or simply more visible or vocal about their ideas than individuals with a mental representation close to attractor-0. This might be because, say, attractor-1 is the scientific explanation that is deemed correct in a particular society. Therefore, when individuals pick a model from whom to copy, there is a higher probability that they will select one closer to attractor-1.

It should be clear from this description that there is only one possible outcome of the model, and that, irrespective of the influence of attractor-0, the entire population will converge on attractor-1. Imagine that the attractors have the same influence: individuals who copy models who are closer to attractor-0 will move, because transmission is imperfect, even closer to attractor 0, and likewise individuals copying models closer to attractor-1 will move closer to attractor-1. However, any selective force, however weak, favouring a tendency to copy models closer to attractor-1 will bring the population to that equilibrium. Even when attractor-0 (the non-selected one) is disproportionally stronger in terms of cultural attraction than attractor-1, as long as at least one individual is in the “area of influence” of the latter, the population will converge to attractor-1. In other words, selection trumps attraction.

The conclusion of the model is, therefore, that when both selection and attraction are present, the system can still be described by standard discrete replicator dynamics, and selection will always determine the result, no matter how strong is attraction (actually, the stronger attraction is, the better the approximation with replicator dynamics) and no matter how strong is the non-selected attractor.

Claidière and Sperber (2007) criticised Henrich and Boyd’s model, noting that it rests on some problematic assumptions, two of them of primary importance. The first is that the target of selection coincides with one of the attractors. Imagine instead that in a particular society, “mixed” theories of moon behaviour are particularly praised and are favoured by selection, while the pure-scientific and the pure-intentional are still the attractors. What will happen in this situation? In this case, the outcome will be determined by selection *and* attraction. Depending on where the selective peak is in the attractors’ areas of influence, the population will converge on one of the two attractors. If the selective peak is in the area of influence of attractor-1 the population will converge to attractor-1 (not to the selective peak), and the same for attractor-0.

Second, Claidière and Sperber (2007) point out that attractors in Henrich and Boyd (2002) model are deterministic. Individuals will end up, no matter what, in an attractor once they are in its area of influence, and this is the reason why non-preservative cultural transmission can be reduced to replicator dynamics. In contrast, they present a notion of probabilistic attraction, for which, depending on the force of attractors, individuals can actually escape from their influence (a *weak* attractor in Henrich and Boyd (2002) model is just an attractor that requires a longer time for the population in its area of influence to converge). While probabilistic attraction basically entails adding noise to the original model, it shows that, in this more realistic situation, the relative strength of attraction and selection are important for the outcomes, and both can contribute to cultural evolutionary dynamics.

While the models hint at some interesting questions that could be explored experimentally (e.g. how likely is selection to coincide with attraction in real life dynamics? How noisy is transformation in cultural transmission?), this exchange shows that it is difficult, using thought experiments or modelling alone, to settle the question. The argument from Claidière and Sperber (2007), which suggests a variable role of attraction and selection in determining the outcome of cultural evolution, seems intuitively more convincing. However, the role of preservative and reconstructive processes in cultural transmission is ultimately an empirical question. In the next sections we will thus first examine how reconstructive cultural transmission has been studied in recent experimental research in cultural evolution. Then, we will discuss how the relative importance of preservative and reconstructive processes may depend on the granularity of the analysis (i.e. on what one considers as the unit of analysis, a “cultural trait”) and how it varies in different domains.

## Empirical evidence relating to the study of preservative and transformative processes

### Experimental studies of cultural transmission

Even though transformation is most commonly associated with the cultural attraction approach, transformation is actually a common subject of study within the standard cultural evolution literature. For example, Mesoudi and Whiten (2004) examined the transformation of event knowledge (descriptions of everyday events such as going to a restaurant) as it is passed along chains of participants each of whom receives the previous participants’ recall as their input. In line with schema theories from cognitive psychology, it was found that low-level actions (e.g. ‘he opened the door, took a seat at the table) were spontaneously subsumed into medium-level (e.g. ‘he entered the restaurant) and high-level (e.g. ‘he went to a restaurant) goals, despite the latter being absent from the original description. The driver of change here is clearly individual modification, which occurred similarly and independently in all participants based on previously acquired and common knowledge structures. While not described as (narrow) cultural attraction in the paper, it is clearly an example of it.

In the same way, although they do not use the term ‘cultural attraction’, experiments and models in the Bayesian inductive reasoning or iterated learning tradition (Kirby et al. 2007; Griffiths et al. 2008) also appear to capture the kind of change envisioned by cultural attraction proponents. For example, Xu et al. (2013) experimentally simulated changes in colour labels, noted by Claidière et al. (2014) to be potentially constrained by psychophysical aspects of cognition that may form a cultural attractor. Xu et al. had participants learn to pair novel words with specific colour shades. While the first participant learned random word-colour pairings, subsequent participants were trained in the word-colour pairs produced by a previous participant, along chains of 13 cultural ‘generations’. Each participant made non-random changes such that the fictional terms gradually converged on real-life colour term clusters. Similar iterated learning experiments have shown that properties of languages, such as compositionality, spontaneously emerge as participants individually modify artificial languages to make them more learnable (Kirby et al. 2008). The source of change in all these cases lies in individual participants’ cognition and perception, which act in similar ways across people to drive cultural representations towards similar end-points, in line with the notion of cultural attraction.

Other studies have combined guided variation and selection-like biased transmission. Bettinger and Eerkens (1999) found that different patterns of prehistoric arrowhead variation in North America showed signatures of different mechanisms of cultural transmission. Arrowheads from Nevada showed little variation, coming in a small number of uniform types. Contemporary arrowheads from California showed extensive variation, with no uniform types. Bettinger and Eerkens (1999) argued that the former pattern was generated via model-based biases, with prehistoric hunters preferentially copying the arrowhead designs of successful or prestigious hunters, thus creating a small number of popular types. The more diverse Californian arrowheads, on the other hand, were influenced by guided variation, as each arrowhead maker modified their design according to trial-and-error. Mesoudi and O’Brien (2008) subsequently experimentally simulated these hypothesised transmission processes, confirming that model-based bias and guided variation can, under certain circumstances, generate the observed patterns of low and high variation respectively. This general hypothesis is noteworthy because the individual modification component is not a result of common content-based cognitive biases, but instead due to contentless trial-and-error (associative) learning.

In the only experimental study that we know of that has explicitly compared cultural attraction and selection, Eriksson and Coultas (2014) examined the transmission of stories that invoke to varying degrees emotional reactions of disgust. In one experiment they passed stories along chains of participants in the standard manner, finding that elements rated highly disgusting were preserved over elements rated low in disgust. This can be seen as a form of cultural attraction, with the stories mutating at each step in a non-random direction to contain relatively more disgusting content. In a further experiment, Eriksson and Coultas (2014) allowed participants to choose whether to read, and then whether to pass on, a story to a subsequent participant, without altering the story. Hence this resembles selection (specifically, content/direct bias), because the stories change in frequency without being altered. Both methods revealed a bias towards disgusting content, indicating that disgust bias operates both through the non-random transformation of content as it is remembered and reconstructed, and also the non-random selection of content as it is chosen and replicated. Although Erikkson and Coultas did not discuss this, it appears that selection had a larger effect, in that low-disgust material was entirely absent at the end of the chains in the experiment in which only selection was possible, whereas in the experiment in which only transformation was possible, low-disgust material was still present at the end. In explaining the real-life preponderance of disgusting urban legends (see Heath et al. 2001), both cultural attraction and cultural selection can potentially be seen to be working together.

Here are some take-home messages from these studies. First, cultural attraction, transformation and cognition are not ignored in standard cultural evolution research. Many studies, in particular transmission chain studies, have explicitly examined transformative processes. If anything, it is rarer to find transmission chain studies that examine cultural selection. Second, few studies have explicitly studied both selection and attraction. Eriksson and Coultas (2014) is a rare exception. Third, studies such as Mesoudi and O’Brien (2008) highlight that cultural attraction does not have to be due to cognitive universals. The individual modification that occurs in cultural attraction can occur via individual trial-and-error. If a task has multiple solutions, then perhaps trial-and-error will lead different people to different solutions (as it did in Mesoudi and O’Brien’s study), such that cultural attraction can generate and maintain cultural diversity.

### Preservative versus reconstructive processes depend on the granularity of the analysis

In some cultural evolution studies, the unit of analysis is the cultural trait, that is, what is transmitted in the cultural transmission process. Examples of cultural traits include names, fairy tales, ways to tie a knot, recipes for lasagne, hammers, and the like. In others, the unit of analysis is the individual person (see also El Mouden et al. 2014). If each individual has exactly one cultural variant of a particular type, then these units will coincide. However, where individuals can possess multiple cultural traits, then classifying cultural change as attraction or selection-like becomes complicated. Image that person A has ideas X, Y, and Z, and person B learns from A only ideas X and Y, with no modification of those traits. From the trait-as-unit-of-analysis perspective, transmission is preservative: traits X and Y are being selected and transmitted with high fidelity, while trait Z has been selected against. From the individual-as-unit-of-analysis perspective, however, transmission may be considered reconstructive, as person B has a different set of traits (XY) compared to person A (XYZ), from whom she copied.

Take, for example, a transmission chain experiment by Mesoudi et al. (2006), in which multiple stories varying in their social complexity were passed along chains of participants. Over successive transmission episodes, the social stories remained largely intact, while the non-social stories virtually disappeared. If one takes the individual as the unit of analysis, then this appears to be a case of cultural attraction. The first people in the chains had a mixture of social and non-social stories, the final people had mostly social, such that there is a non-random transformation due to (according to Mesoudi et al.) biologically evolved and universal aspects of cognition (humans’ ‘social brains’). If instead one considers each separate story as a ‘trait’, then the process seems more selection-like. The social stories were more likely to be preserved, and the non-social stories less likely to be preserved, with no modification to the traits (there was little distortion or confabulation in this particular study). This change in trait frequency therefore resembles selection. Note that there is no explicit, conscious ‘selection’ of stories by the participants here, just unconscious selection as a result of (probably implicit) memory biases; the population level consequences of both explicit/intended and implicit/unintended selection will be the same, however. In sum, there doesn’t seem to be a ‘correct’ answer to whether people or traits are the unit of analysis, but which decision we take determines whether the process is transformative (attraction-like) or preservative (selection-like).

The issue regarding preservation and transformation in transmission, however, is generally considered assuming the trait-as-unit-of-analysis perspective. Consider again Cinderella. We used it above as an evident case of reconstructive cultural transmission since, each time one retells the story, it will be extremely unlikely that she will repeat exactly the version heard. However, what are we considering here as the cultural trait? A coarse-grained description of the cultural trait is “a story involving a young lady, first oppressed by her stepmother and stepsisters, and then succeeding in marrying a prince”. Because this basic plot structure is likely to be maintained through successive iterations, the transmission is, at this level, preservative. At an intermediate level we can consider, for example, Cinderella as a combination of sentences. In this case, assuming that one repeats all the sentences, one might change some words, saying: “Once upon a time there lived a sad young girl” instead of “Once upon a time there lived an unhappy young girl”. This would count as reconstructive. Finally, a fine-grained description could focus on the single words of the story. Imagine one summarises Cinderella in few sentences, using words picked from the perhaps longer version she heard. One could interpret this as a preservative process, in which some cultural traits (the words used) have been selected and reproduced without mutation.

Moreover, cultural selection and cultural attraction are likely, in the majority of cases, to act together within the same traits, at different levels of generality. As we mentioned above, supernatural concepts may be favoured because they are minimally counter-intuitive entities. As an optimal combination of intuitive and counter-intuitive features, a generic undead being (like a ghost, or a zombie, or a vampire) is an effective cultural trait. However, an explanation of the cultural success of a specific undead entity, say, Dracula, needs to include selective processes. The spreading of Dracula is most likely due *both* to attraction-related factors, that explain why, in general, undead beings are favoured in respect to other entities, *and* to selective factors, that explain why, among all other undead beings, the Transylvanian vampire enjoys such popularity.

One could hope that, when we find the *correct* unit of analysis for cultural evolution, we would be able to settle the debate. Unfortunately, this might be unlikely, as there is continuing disagreement over how to define a cultural trait. Sperber and Claidière (2008) criticize Richerson and Boyd (2005) for seeming to oscillate between an “internalist” view of cultural traits as “(mostly) information in brains” (*ibidem*, p. 61) and an “externalist” one where “some cultural information is stored in artifacts” (*ibidem*, p. 61). However, the cultural attraction approach adopts a similar strategy assuming that both mental representations and behaviours/artifacts should be considered cultural traits (Sperber and Claidière 2008). Claidière et al. (2014), for example, explicitly discuss how mental representations and public narrations of a folktale should be both treated as cultural traits with “equally potent causal roles” (*ibidem*).

One proposed solution to this puzzle is to consider the information, wherever stored, as the equivalent of the biological genotype, and the expression of the information in behaviours or artifacts as the equivalent of the biological phenotype (Dawkins 1976). The problem here is that it assumes that, when copying, we have access to a “cultural core” (Sperber and Claidière 2008), which represents the information/genotype, which we then use to build variable phenotypic expressions. This might be loosely the case: the classic example is the transmission of a recipe to cook, say, lasagne, where the recipe represents the transmitted, stable, genotype, and what you serve to your guests at dinner is the variable phenotype. However, in many cases, we do not have access to a “recipe”, but we extract the information from the result/phenotype (such as when we try to reproduce lasagne after tasting it at a friend’s home). Richerson and Boyd (2005) make a similar point when noting how the mental representations of different individuals who have tied the same bowline knot might in principle be very different. What is the genotype here? The individual, variable, mental representations of the bowline knot cannot be the genotype, as they are not, in general, transmitted, because they are different. For the same reason, the information stored in the artifact itself does not transfer directly in the (variable) mental representations.

Furthermore, even solving the internalist/externalist debate would not settle the reconstructive/preservative question. Imagine that everybody agreed on an internalist view, so that the *real* cultural traits in the transmission of a folktale are the mental representations, which we could access with some advanced neuroimaging technique. As we suggested in the Cinderella example, would they be the mental representations of “a story involving a young lady, first oppressed by her stepmother, etc.” or more detailed mental representations of the plot, or something else?

While this may appear pessimistic, we believe that pluralism in the conceptual definitions of the unit of analysis in cultural evolution is not a problem (see also Lyman and O’Brien 2003; O’Brien et al. 2010). Biologists, too, work simultaneously with multiple concepts of the ‘gene’, varying with context and use (Stotz and Griffiths 2004). Depending on various domains, and on the questions one is interested in, an opportunistic strategy can be the best choice. Moreover, moving from coarse to fine grained units can indeed clarify how the interplay between attraction and selection can be important for the success of specific cultural traditions, as the Dracula example illustrates.

### Preservative versus reconstructive processes depend on the empirical domain

Besides the decision of what to consider a cultural trait, the fidelity of cultural transmission likely also varies in different empirical domains. We initially compared two cases. In the oral transmission of stories, we can infer from high variability of the successive reproduction of the “same” story that reconstructive processes strongly influence cultural evolution. In first names diffusion, instead, the innovation rate is extremely low, and cultural transmission is highly preservative, such that selective processes are more important than attraction-based processes. Many other examples are possible across the domains of technology, language, art and social customs.

The general scepticism of proponents of cultural attraction towards the idea that high fidelity imitation is the unique, or even the main, support for cultural evolution is a useful counterbalance to a naïve view of humans as perfect and indiscriminate copy-machines, and that this is enough to explain cultural stability. Not only are copying mechanisms often characterised by low fidelity (as in the Cinderella example), but also long-term, stable, traditions are not necessarily supported by high fidelity copying (as in the religion example, where supernatural concepts may be reconstructed each time). However, it does not follow from here that copying mechanisms are *always* scarcely faithful, or that stable traditions are *never* supported by high fidelity copy.

Many technologies that we use are, for example, causally opaque (Csibra and Gergely 2011), meaning that we do not know or understand the mechanism by which they produce the result we use them for. Experimental studies have demonstrated how common high fidelity copying is for technology-related actions. Flynn and Smith (2012) had adult participants observe a model perform some operations with a box (using a tool to drag some bolts, tapping with the tool, lifting a door, inserting a tool into a hole) in order to retrieve a reward from inside. Only the last two of these actions actually retrieved the reward, the others had no causal effect in relation to the goal. One group of participants observed the model interacting with a transparent box and were thus able to see which actions were unnecessary. For another group the box was opaque, obscuring which actions were causally relevant. Flynn and Smith (2012) found that adults, like children (Lyons et al. 2011), showed a high likelihood of copying all actions—both relevant and irrelevant—under both conditions, even the transparent condition where the irrelevant actions are revealed to be irrelevant. This phenomenon, dubbed ‘over-imitation’, indicates that high fidelity copying is often the default approach to solving unfamiliar problems, even out-weighing causal reasoning. Interestingly, however, when the model was another participant (rather than the experimenter) Flynn and Smith (2012) found that participants did *not* reproduce the unnecessary actions when the box was transparent (they still did when the box was opaque). In other words, when potential sources of prestige are removed (thus removing the possibility of prestige biased cultural selection), a causally transparent technology elicited reconstructive transmission, while a causally opaque technology elicited preservative transmission. More generally, we suspect that the more a technology is opaque, the more cultural transmission will be preservative; the more a technology is transparent and model-based biases are absent, the more cultural transmission will be reconstructive.

Other domains that might be characterised by generally preservative transmission are domains in which a final result is reached through a sequence of actions, and sequences of actions that are even slightly different to the correct one produce an unusable result (Acerbi et al. 2011). Tying a Windsor knot is a serious affair that involves a sequence of precise actions. Performing correctly, say, nine of the ten actions required does not produce 90 % of a Windsor knot, but will likely produce a shapeless configuration of fabric. The task of tying a Windsor knot can be visualised as a search space with a single slender peak (the correct knot) surrounded by a vast flat territory (all the action combinations that produce unusable results). For these tasks, individual learning—or reconstruction—is in general an unsuccessful strategy, because the final result does not provide any feedback about “how close” one is to the correct solution, nor has genetic evolution provided us with precise intuitions about knot-tying. Individual learners need to explore each time the full space of possible actions. The great majority of modern technological tasks probably fits this description. While constraints that can help individual search and reconstruction do exist—an airplane has to fly and a kayak has to float—their guidance is so loose that only preservative cultural transmission can sustain those traditions (Acerbi et al. 2012). Notice that examples of opaque or slender-peaked tasks are not necessarily restricted to the technological domain. Other activities that require performing arbitrary but well-defined sequences of actions, like dancing or rituals, could in the same way require preservative cultural transmission to persist (Tennie et al. 2009).

Saying that high fidelity copying is the best strategy in certain situations, or that some traditions need to be supported by high fidelity copying, does not guarantee, of course, that this is what happens in reality. We may indeed use suboptimal strategies, and persistence of traditions can be explained by something else. However, we have good reasons to believe that, *for some domains*, this is indeed the case. Csibra and Gergely (2011) suggest that a suite of species-specific cognitive adaptations for cultural learning, which they label ‘natural pedagogy’, may be responsible for the capacity of preservative cultural transmission of opaque technologies. Natural pedagogy indicates that social learning is accompanied by ostensive communication, that is, a form of deliberate communication (“Look at what I am doing with this stick!”) that guides the learner through the critical aspects of the process. Similarly, Herrmann et al. (2013) showed that verbally framing a demonstration stressing the conventionality of the actions involved (as opposed to their instrumentality) is sufficient to increase imitative fidelity in preschool children. Others (Tennie et al. 2009) have emphasised how high fidelity in human cultural transmission can be achieved through a combination of process-oriented imitative social learning (humans tend to pay attention not only to the final result of a demonstration—a Windsor knot—but also to the actions performed to reach the result) and a form of cooperation that favours active teaching and social motivations to copy. The afore-mentioned over-imitation studies, where people copy both relevant and irrelevant actions demonstrated by others, provides evidence for this.

Another important factor that increases the fidelity of cultural transmission is the use of epistemic tools (Sterelny 2006). Epistemic tools are modifications of the environment—in a broad sense—that improve the cognitive capacities of individuals. Tasks that are hard for children to learn, such as tying their shoes, can be encoded in vivid images and rhymes such as “Bunny Ears”. Tehrani and Collard (2009) argue that they are able to trace robust phylogenies (a sign of preservative transmission) of Iranian tribal textiles because craft learning is scaffolded in such a way that different designs are embodied as a set of motor routines that are difficult to rewire. Modern culinary recipes are another good example of epistemic tools. They convey detailed information through numbered lists of ingredients, with universal measures, explicit sequences of actions, and possibly images of the various phases of the preparation. As with all technical idioms, cookery language has developed a series of specific terms (to sauté, to simmer, to reduce, etc.) that decrease ambiguity and, again, favour preservative transmission.

Of course, language itself is a preeminent epistemic tool, and written language has been explicitly considered as a technology that favours preservativity of cultural transmission, compared to oral communication (Ong 1982; Rubin 1995). One innovative line of studies examined the hand-copying by scribes of stories before the invention of the printing press, stories such as The Canterbury Tales (Barbrook et al. 1998; Howe et al. 2001). Phylogenetic analyses accurately reconstructed the evolutionary relationships between the different manuscripts due to the high fidelity copying. There were also copying errors intriguingly similar to those found in genetic inheritance, such as the insertion or deletion of words or letters, or the random swapping (or ‘crossing over’) of sentences from one manuscript to another. In these cases, where the express goal is to replicate a text, there was seemingly very little directional transformation.

Today, we can observe a new shift that involves digitally mediated cultural interactions. The transmission of Internet content (think of social media “sharing”) is a form of highly preservative cultural transmission, where the information is practically replicated with no mutation. Intriguingly, there are several examples of short texts that have become “viral” in social networks such as Facebook or Twitter which users are explicitly asked to not automatically share or re-tweet, but to copy and paste manually (Adamic et al. 2014). This re-introduces the possibility of transmission errors or conscious modifications, or, in other words, makes transmission more reconstructive in a preservative media like the Internet. Adamic et al. (2014) found indeed a decrease in transmission fidelity (a mutation rate of 11 %), with some non-random modifications. For example, the phrase “No one should die because they can’t afford health insurance…” was transformed by conservatives into “no one should die because the government is involved with health care…”, reminiscent of Bartlett’s (1932) early studies where information is distorted to fit pre-existing opinions. Overall, it is a fair question to ask whether the ubiquitous presence of digital communication is making cultural transmission more preservative than reconstructive and what the consequences are of this transformation.

All these examples show that it is important to not automatically assume that human culture is sustained by perfect transmission, but how, in some domains, the fidelity of cultural transmission is higher than in others. Rather than deciding whether attraction or selection is *in general* more important, it is more interesting to ask the extent to which transmission is preservative or reconstructive in different domains, and how attraction and selection consequently interact to shape cultural variation.

## Discussion

In this paper we examined a recent debate in the field of cultural evolution. Our impression is that this debate is partly driven by confusion among the practitioners, so we tried to clarify areas of genuine disagreement and of common ground, and, especially, to elucidate how the issues have been, or could be, examined in empirical studies. Our main message is that there is no insurmountable theoretical disagreement between the standard cultural evolution approach and the cultural attraction approach, but rather a focus on different aspects of cultural transmission and evolution.

Specifically, we think that it is useful to distinguish between a broad definition of the “attraction” concept (as described by Sperber 1996 and Claidière et al. 2014), where attraction encompasses *any* directional process in cultural evolution, and a narrow definition (more similar to the one modelled in Claidière and Sperber 2007), where attraction is contrasted to selection, and relates specifically to transformative and constructive processes in cultural transmission. While the broad concept of attraction has the advantage of being more general (Claidière et al. 2014), it seems to unnecessarily replace a series of concepts, such as direct and indirect biases, which have already been formally and extensively modelled and in many cases have strong empirical support. Moreover, generality is not necessarily better. For example, as population geneticists can reasonably assume high-fidelity transmission, they have the advantage of being *less* general. The need for generality in cultural evolution is not good per se, but should be associated with the relative importance of transformative and constructive processes. Like others (e.g. El Mouden et al. 2014), we see purely preservative and purely transformative processes in cultural transmission as two extremes of a continuum, and therefore the relative importance of selection and attraction in cultural evolution should be an empirical problem, depending on the domain studied, on what one considers a cultural trait to be, and, ultimately, on what level of explanation one is interested in.

A narrow definition of attraction usefully points to the importance of transformative and constructive processes in cultural transmission, but, we argued, there is no theoretical disagreement between the two approaches. Standard cultural evolution models, from the very beginning, have contained transformative processes such as guided variation, that seem to us to be identical to narrow cultural attraction. It might be argued that standard cultural evolution has focused more on modelling preservative processes, and selection among traits, but it is unfair to characterise the standard cultural evolution approach as having ignored transformative processes.

A focus on the differences between transformative and preservative processes in cultural transmission suggests empirical studies that could be done to move forward cultural evolution research. We noticed how, with rare exceptions (Eriksson and Coultas 2014), virtually no studies in the cultural evolution tradition had directly dealt with this challenge. In addition, in Eriksson and Coultas (2014), selection and attraction worked in the same direction, both favouring stories eliciting an emotional reaction of disgust. It would be informative to explicitly pit different biases against each other and test their relative strength in a standard transmission chain experiment (hence characterised by transformative transmission) versus a simple task of choosing which story to pass on (preservative). One may hypothesise, for example, that biases (or “factors of attraction”) related to memorisation and verbal reproduction would be more favoured in the transformative than in the replicative condition, where perhaps contrasting biases related to “classic” cultural selection (for example a prestige, or conformist, bias) could overcome the strength of the former.

Additionally, while lab experiments are useful for showing what kind of biases are possible, they cannot tell us which process is responsible for specific real-life cases of cultural change. The analysis of real-life historical or cross-cultural datasets is thus of primary importance for determining the relative role of attraction and selection in cultural evolution. Morin (2013), for example, proposed that direct eye gaze, as opposed to averted eye gaze, is a potent cultural attractor with respect to portrait aesthetics, due to “our innate propensity to look at direct-gaze faces” (p.227). He showed that (1) direct gaze portraits are more likely to be reproduced in art books, (2) direct gaze portraits increased in popularity over time, and (3) individual painters did not modify their style over their lifetimes to prefer direct gazes, instead the population-level change is driven by new generations of painters who had stronger direct eye gaze preferences replacing earlier generations. He therefore proposed that new apprentice painters either transform the style of their teachers towards the direct eye gaze attractor (cultural attraction), or apprentices are selectively exposed to the most popular paintings of the time, which were direct eye gaze paintings, or selectively copy paintings that happened to have direct eye gaze (cultural selection/biased transmission). Morin (2013) concludes that “a combination of cognitive attraction, cultural selection and demographic turn-over seems a promising explanation” for the phenomena in question. More fine-grained, individual-level data may be able to distinguish between these alternatives.

We briefly mentioned, at the end of Sect. 3.3, how present-day digital interactions, besides representing an extraordinary source of quantitative data to study cultural evolution, provides an epistemic tool for highly preservative cultural transmission. Accordingly, it might be interesting to analyse whether and how the preservativity of such tools influences the strength of different biases, compared, for example, to an analogous orally transmitted tradition. More generally, the availability of large amounts of quantitative data in digital form could be used to estimate precisely the degree of transmission fidelity (as in Adamic et al. 2014) and to compare it in different areas, giving some indication of where to place different cultural domains along the preservative/transformative continuum. Phylogenetic methods are also being brought to bear on this issue with increasingly detailed historical quantitative data being used to determine the transmission fidelity of different folk tales (Tehrani 2013) and words (Pagel et al. 2007), for example.

In conclusion, we think that an interest in different aspects of cultural transmission and evolution, far from representing a deadlock for cultural evolution studies, can inspire new empirical studies and draw attention to details of transmission not yet explored. We hope that this paper has gone some way to clarifying potential points of confusion, and highlighted the extent of genuine agreement on the key issues.
